# Balance, Sensorimotor, and Cognitive Performance in Long-Year Expert Senior Ballroom Dancers

**DOI:** 10.4061/2011/176709

**Published:** 2011-09-25

**Authors:** Jan-Christoph Kattenstroth, Tobias Kalisch, Izabela Kolankowska, Hubert R. Dinse

**Affiliations:** ^1^Neural Plasticity Lab, Institute for Neuroinformatics, Ruhr-University Bochum, 44780 Bochum, Germany; ^2^Department of Neurology, BG-Kliniken Bergmannsheil, Ruhr-University Bochum, 44789 Bochum, Germany

## Abstract

Physical fitness is considered a major factor contributing to the maintenance of independent living and everyday competence. In line with this notion, it has been shown that several years of amateur dancing experience can exert beneficial effects not only on balance and posture but also on tactile, motor, and cognitive functions in older people. This raises the question of whether an even more extensive schedule of dancing, including competitive tournaments, would further enhance these positive effects. We therefore assessed posture, balance, and reaction times, as well as motor, tactile, and cognitive performance in older expert ballroom dancers with several years of competitive experience. We found substantially better performance in the expert group than in the controls in terms of expertise-related domains like posture, balance, and reaction times. However, there was no generalization of positive effects to those domains that were found to be improved in amateur dancers, such as tactile and cognitive performance, suggesting that there might be an optimal range of intervention intensity to maintain health and independence throughout the human lifespan.

## 1. Background

In addition to a general decline in physical fitness [1], the aging process is accompanied by a progressive decline in perception, motor behavior, cognition, and memory functions [2–4]. Therefore, the preservation of everyday life skills and the maintenance of independent living become increasingly important with advancing age. It is well established that physical fitness is intimately associated with cognitive performance in the elderly [5–9]. Consequently, high levels of physical fitness have been assumed to be a major factor contributing to the maintenance of independent living and everyday competence. 

One of the basic accomplishments of gerontology is the recognition of the tremendous heterogeneity and interindividual variability in the elderly [10]. Thus, the emergence of age-related decline can be highly variable between individuals [2], and there are notable differences in the interindividual performance of general skills at advanced ages. It seems that, aged individuals can maintain high levels of proficiency in certain domains involving cognitive-motor functions such as golf or piano playing. This gave rise to an intriguing question: how is proficiency in one domain of expertise, like playing piano, associated with performance in general? (for review, see [4]). Older experts show little or no age-related decline in tasks related to their area of expertise, but beyond that they show a general age-related decline similar to the nonexpert older adults [10]. On the other hand, the maintenance of high levels of expertise in one domain can have a positive impact on related functions. For example, elderly professional pianists have higher finger-tapping rates than untrained aged-matched individuals [11]. 

We recently showed that a regular schedule of amateur dancing over many years throughout old age not only promotes posture and balance, but also has a wide range of beneficial effects on reaction times (RTs), motor behavior, and tactile and cognitive performance by comparing such individuals with an aged-matched nondancer control group [12]. We hypothesized that the generalization of superior performance associated with regular dancing develops as a result of physical exercise in combination with cognitive challenges, sensory stimulation, and social interaction, all of which contribute to neuroplasticity. 

Here, we extended these studies by investigating the impact of dancing at a higher level of expertise. One of the rationales for this study was to obtain information about whether a more extensive schedule of dancing, including competitive tournaments, would further enhance the range or magnitude of beneficial effects. We compared a group of neurologically healthy older subjects with many years of expert and competitive experience of dancing (ED) to a gender-, age-, and education-matched nondancer control group (CG). In this study, the term “expert” is defined as those who regularly attend dance competitions and dance contests and undergo training at intensities of more than 4 h/week. Comparable to our previous study on amateur dancers, we measured posture and balance, cognitive, attentional, intellectual, perceptual, and sensorimotor performance.

## 2. Materials and Methods

### 2.1. Subjects

A total of 49 healthy volunteers (60–94 years) participated in our study. Subjects were recruited by advertisements in newspapers, poster announcements, and word-of-mouth advertising. All subjects reported their medical history and current medication and underwent the Mini-Mental Status Examination (MMSE) [13]. The ED group (*n* = 11, 5 women, 71.18 ± 1.13 years) had an extended history of competitive dancing (22.09 ± 3.39 years) with an average workload of 4.55 ± 0.15 h/week. Subjects in the ED group reported a regular attendance in official dance contests and championships throughout Germany. The CG group consisted of 38 sedentary subjects (71.66 ± 1.11 years, 30 women, ECQ score: 8.43) with no record of dancing or sporting activities (see Section 4 for details regarding the selection of appropriate controls). The age distribution (*P* = 0.829) and education level (number of school years, *P* = 0.926) of subjects across the groups was balanced. All subjects gave their written informed consent before participating in the study. The study was approved by the local Ethics Committee of the Ruhr-University of Bochum.

### 2.2. Competitive Ballroom Dance

During dance competitions, all subjects in the ED group were assigned to a starting group referred to as “seniors IV” (age > 66 years). For the competition, 10 different dances had to be performed in a mandatory order, including the slow waltz, tango, Viennese waltz, slowfox, quickstep, samba, cha-cha-cha, rumba, paso doble, and jive, each of which lasted for 1.5–2 min. On the basis of points given by adjudicators during the contests, the subjects of our ED group were assigned the highest German grade (S) within the corresponding starting group. Therefore, subjects of the ED group had to be particularly fit with regard to mobility, muscle flexibility, and body composition. Although literature reports indicate a lower cardio-respiratory performance (i.e., maximal oxygen uptake or VO2_max_) for professional ballet dancers in comparison to other athletes performing physical activities like running or swimming, professional modern dancers were shown to have a significantly higher maximal oxygen uptake compared to professional ballet dancers (for review, see [14]). 

Given the average workload of 4.5 h/week plus assumed 2.5 h/week for dancing competition adds up to 7 h a week, which totals 350 h per year, which sums up to 7500 h, which is the typical workload range required to qualify for becoming expert [15].

### 2.3. Everyday Competence

Lifestyle and general activity levels were assessed using the Everyday Competence Questionnaire (ECQ) [16] that addresses the aspects of everyday life, such as independence in activities of daily living and mobility, social relations, general health status, and life contentment. The compilation of questions used in the ECQ accounts for the changing living conditions of today's seniors. The ECQ consisted of 17 items, including housekeeping, daily routines, manual skills, mobility, sports, subjective well-being, linguistic abilities, and leisure-time activities [17], thereby addressing instrumental activities of daily living (IADL) [18] and the individual engagement in other activities of everyday life as well. These activities are not necessary for fundamental functioning, but they let an individual live independently in a community. 

All subjects were asked to comment on the questions with as much detail as possible, thus allowing insight into their habits and living conditions. The answers were converted into numerical scores according to an item-specific scale. Altogether, subjects could achieve 0–54 points. The scores were normalized to a scale from 0 to 1 by dividing the number of points achieved by the maximum possible scores per item. For a detailed description, see [16].

### 2.4. Cognitive Performance

**Figure 1:**
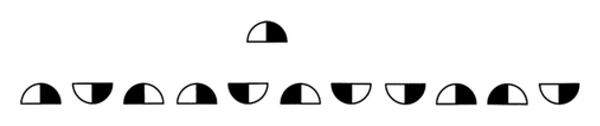
Single row of the nonverbal geriatric concentration test (AKT). Subjects had to mark 20 symbols equivalent to the one at the top in five rows of 55 similar looking patterns within a maximum time limit of 30 s. After an initial training session, three consecutive sessions were run. Needed times for each session were averaged for evaluating individual performance.

Based on figural reasoning, general intelligence was assessed using the Raven's Standard Progressive Matrices (RSPM) [19, 20]. The test was administered according to standard instructions with a 30 min time constraint. In the control group, the RSPM was conducted in a pre-/post-design to provide data for a separate study. Therefore, for both groups in this study, we used odd-numbered items only, resulting in a maximum score of 30. In addition, the nonverbal geriatric concentration test (AKT) [21] was used to assess selective attention and concentration. For this paper-and-pencil test, subjects had to mark 20 symbols of 55 similar-looking patterns within a maximum time limit of 30 s (Figure 1). After an initial training session, 3 consecutive test sessions were performed. The time required for each subject to complete the test sessions was averaged to evaluate individual performance.

### 2.5. Multiple-Choice Reaction Time Measurement

We performed multiple-choice RT measurements in a finger-selection visuotactile task adapted from the study of Alegria and Bertelson [22]. Subjects were seated 3 m in front of a monitor. An image of each hand was displayed on the monitor and 1 finger of the 10 was selected by a visual marker. Subjects had to press the key corresponding to the selected finger on a hand-shaped, 10-button keyboard as fast as possible. One session consisted of 4 blocks of 100 trials each, which were separated by a short break after each block. The maximum response-to-stimulus interval for each trial was 2000 ms. Each finger was tested 40 times in a random order.

### 2.6. Posture, Balance, and Gait Control

We applied the *Romberg test* [23], the *timed up and go test *[24], and the *standing-turn test* [25] to assess each subjects' ability to control their posture and maintain balance and to evaluate their security of gait. The *Romberg test* is a standard neurological test addressing joint position sense (proprioception) and was applied in a condition with eyes either open or closed [23, 26]. The subjects were asked to stand upright with their feet in a tandem stance. The movements of the body in relation to a perpendicular object behind the subject were monitored. A second experimenter stood close to the subject to prevent the person from falling. The time until a subject started to lose balance was recorded (maximal testing time was limited to 1 min). In the *timed up and go test*, subjects were asked to stand up from a sitting position, walk 3 m, return to the chair, and sit down again. The time to fulfill the task was measured. In the *standing-turn test*, a standing subject was asked to perform a 360-degree turn. The time and number of steps were documented.

### 2.7. Motor Performance

Hand-arm fine-motor performance was evaluated using a computer-based test battery for clinical neuropsychological research (MLS; Dr. G. Schuhfried GmbH, Mödling, Austria). The system consists of a work plate with 2 pencils for left and right hand use. We tested speed, accuracy, and maintenance of upper limb position during execution of fine motor movements of the left and right arms, hands, and fingers by using the following tests for: *steadiness*, which evaluates the ability to achieve a prescribed arm-hand position and maintain it for a defined time period; *aiming*, which evaluates the ability to accomplish fast arm-hand movements for small targets; *pin plugging*, which evaluates fine and gross motor dexterity and coordination; *tapping*, which evaluates the ability to perform very fast, repetitive wrist-finger movements with little emphasis on the precision of movement.

### 2.8. Tactile Performance

#### 2.8.1. Touch Threshold

Touch threshold was evaluated using a staircase procedure by probing the fingertips of the left and right index fingers with von Frey filaments ranging from 0.25 to 294 mN on logarithmic scaling (Marstocknervtest, Marburg, Germany).

#### 2.8.2. Two-Point Discrimination Threshold

Spatial 2-point discrimination thresholds (*2pd*) were assessed on the tips of the left (LID) and right (RID) index fingers by using the method of constant stimuli [27–29]. Needle separations of 1.5, 2.3, 3.1, 3.9, 4.7, 5.6, and 7 mm were used. Test-retest reliability using this procedure was 0.90 for young subjects and 0.88 for older participants [30]. The summed responses were plotted against the needle distances resulting in a psychometric function, which was fitted using a binary logistic regression (SPSS, SPSS Inc., USA). The threshold was taken from the fit where 50% correct responses were reached.

### 2.9. Domains

To pool the data obtained from the various tests, we defined 5 domains covering similar functional categories. “Cognitive performance” comprised data from the AKT and the RSPM. “Tactile performance” comprised data from *touch threshold* and *2pd*. “Posture and balance” comprised data from the *Romberg test*, the *timed up and go test*, and the *standing-turn test. *“Motor performance” comprised *steadiness*, *aiming*, *pin plugging*, and* tapping. *A separate domain “RT” was introduced to include data from the multiple-choice RT task.

### 2.10. Indices of Performance

To compare performances across all tests and all subjects, we calculated normalized performance indices (IPs) for each subject, and each test as (wp-ip)/(wp-bp), where wp is the worst performance of all subjects, ip is the individual performance, and bp is the best performance of all subjects. The best IP is 1, while the worst IP is 0. Indices were subsequently averaged across tasks belonging to a particular domain as described above.

### 2.11. Data Analysis

In all cases, we reported averages and standard error of the mean (SEM). We used the Mann-Whitney *U* test to detect differences between the 2 groups. Moreover, we computed effect sizes according to Cohen's d [31]. To test for differences in the distribution of IPs, we used chi-square statistics. A *P* value of <0.05 was considered significant.

## 3. Results

**Figure 2:**
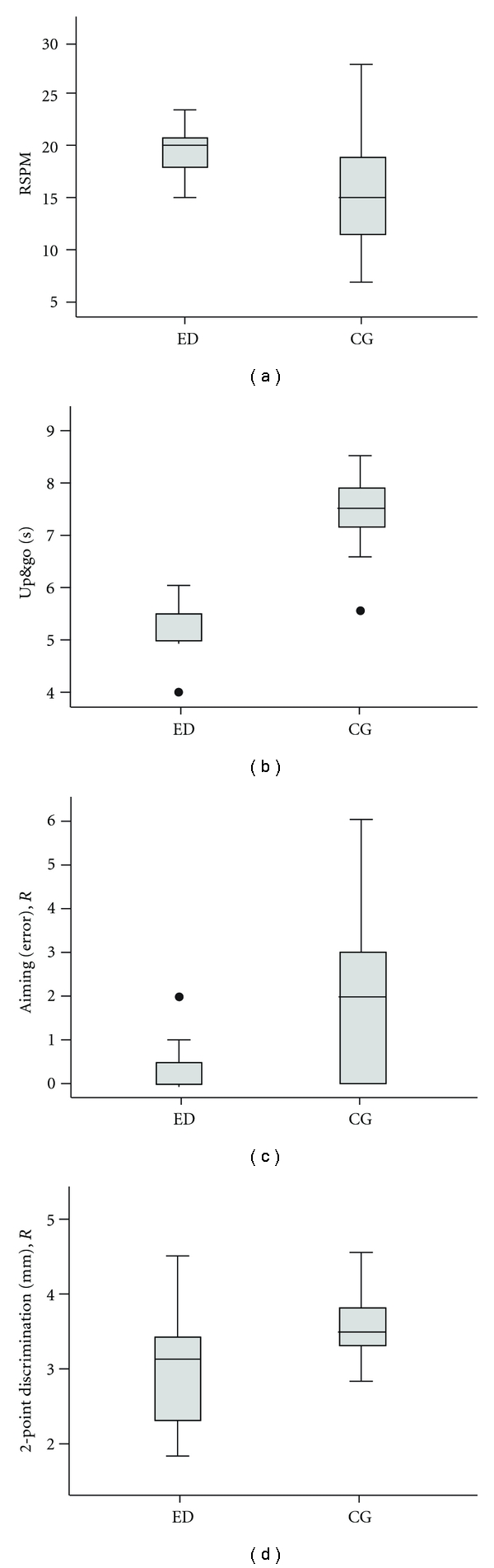
Performance of expert dancers (ED) and a matched control group (CG) for selected tests covering cognitive, posture and balance, motor, and tactile domains. Participants of the ED group showed (a) higher scores in the RSPM (*z* = −2.776, *P* = 0.006), (b) shorter* Up* and *go *times (*z* = −3.819, *P* ≤ 0.001), (c) less errors in the *Aiming* test for the right hand (*z* = −2.808, *P* = 0.005), and (d) lower *2*-*Point-discrimination thresholds* for the right index finger (*z* = −2.434, *P* = 0.015). Horizontal lines within the boxes represent the medians. Boxes show the top and bottom quartiles, and whiskers represent the minima and maxima within 1.5 interquartile range (IQR). Outliers (>3.0 IQR) are labeled as solid dots.

We tested cognitive, posture, balance, and sensorimotor performance in the 2 groups of older participants, matched for gender, age, and education, who had an extended history of expert and competitive dancing (ED), or no dancing experience (control group; CG). The ED group had a superior performance in most of the tests (Table 1). Performance for individual tests is illustrated in Figure 2.

The ED group showed significantly higher ECQ scores than the CG group (*z* = −2.996, *P* = 0.003). Cognitive performance assessment showed significant differences between the ED and CG groups for the RSPM (Figure 2(a)) (*z* = −2.776, *P* = 0.006) and AKT (*z* = −4.997, *P* ≤ 0.001). For both hands, the ED group had faster RTs, which were averaged for the left (*z* = −2.294, *P* = 0.022) and right hands (*z* = −2.195, *P* = 0.028).

Posture and balance assessment showed significant differences between the 2 groups for the *Romberg test* with eyes open (*z* = −3.951, *P* ≤ 0.001), but not with eyes closed (*z* = −1.250, *P* = 0.211). Subjects in the ED group needed less time for the completion of the *standing-turn test *(*z* = −2.815, *P* = 0.005). Moreover, the ED group showed significantly shorter *up and go *times (Figure 2(b)) (*z* = −3.819, *P* ≤ 0.001). Significant differences were found between the 2 groups in the motor domain for the *aiming *subtest (Figure 2(c)), which showed that there were fewer errors in the ED group (*z* = −2.808, *P* = 0.005), as well as a shorter *pin-plugging* time (*z* = −2.343, *P* = 0.019), both of which were observed in the right hand only. In the tactile domain, the assessment of *2pd *thresholds showed significant differences between the 2 groups (Figure 2(d)) for the right (*z* = −2.434, *P* = 0.015) and left (*z* = −2.515, *P* = 0.012) index fingers.

### 3.1. Indices of Performance

The calculation of IP for each test and each subject allowed a direct comparison of performances across all tests and all subjects and facilitated grouping into functional domains covering cognition, RTs, posture and balance, motor performance, and tactile performance. As shown in Table 2, the ED group showed significantly higher IPs in 2 of the 5 domains, with the largest difference being for posture and balance (*z* = −5.599, *P* ≤ 0.001) followed by RTs (*z* = −3.462, *P* = 0.001). 

Our findings indicated a general advantage for the ED group, which spans cognitive, perceptual, and motor performance. In order to obtain insight into possible differences in the overall distribution of IPs within a given domain, we grouped the IP for each domain into >0.5 and <0.5 values and compared the percentage of occurrence of IPs >0.5 across groups, where 0 indicates the worst and 1indicates the best performance.

In 3 of the 5 domains analyzed, the CG group had a significantly higher number of IPs that was lower than 0.5 (subjects with IP <0.5, cognition: ED = 9.09%, CG = 36.26%,  *χ* ^2^ = 6.12, *P* = 0.013; RT: ED = 4.55%, CG = 44.74%, *χ* ^2^ = 12.00, *P* = 0.001; posture and balance: ED = 21.82%, CG = 70.37%, *χ* ^2^ = 25.87, *P* ≤ 0.001). Higher IPs for motor performance that were lower than 0.5 were also found within the CG group, but they did not reach statistical significance (ED = 17.61%, CG = 24.59%, *χ* ^2^ = 3.75, *P* = 0.053). In agreement with the results shown in Table 2, increased IPs lower than 0.5 were found for tactile performance in the ED group (ED = 38.64%, CG = 21.26%, *χ* ^2^ = 5.16, *P* = 0.023). Accordingly, the superior performance of the ED group within some domains did not come from the fact that their best performers were better than those of the CG group, but it was instead due to the fact that the ED group lacked the poor performers that were frequently present in the CG group.

## 4. Discussion

We have recently shown that a regular schedule of many years of amateur dancing in old age has a wide range of beneficial effects not only on posture, but also on sensorimotor and cognitive performance [12]. This observation raised the question of whether preservation of a high level of proficiency such as that which is present in elderly expert ballroom dancers has an even higher positive impact on physical and cognitive fitness in aged individuals as compared to those with only basic amateur dancing skills. We therefore studied the impact of extended participation in competitive dancing in a group of older subjects and compared them to an aged-matched, nondancer CG. In addition to posture and balance, which are closely related to dancing, we performed a broad assessment of cognitive, attentional, perceptual, and sensorimotor abilities. 

According to our hypothesis about the impact of multi-year dancing activities, we expected a broad range of beneficial effects. Therefore, we needed to test many different domains from cognitive functions to basic sensory abilities. Criteria for selecting a test included a brief time needed to complete the test, general acceptance, and a wide extension. In this sense, a particular test served as a surrogate for a given domain, implying that other tests for this field would have shown similar effects. Raven's matrices were selected as a measure of general intelligence. Floor or ceiling effects have been described when using the Advanced (ceiling) or Colored Progressive Matrices (floor effects) [32]. In our study, we used a subset of odd-numbered items only, resulting in a maximum RSPM score of 30. The scores obtained for ED (19.59 ± 0.75) and CG (15.39 ± 0.83) indicate a lack of floor and ceiling effects. 

We included a CG that was characterized by having no record of dancing or sporting activities for at least 5 years. We used the ECQ questionnaire to characterize both cohorts of participants. The ECQ addresses specific aspects of so-called instrumental activities of daily living, such as housekeeping, daily routine, manual skills, mobility, sports, subjective well-being, linguistic abilities, and leisure-time activities. Participants in the CG had lower ECQ scores, indicating a more passive and sedentary lifestyle. These data imply a close association between a lack of sporting activities and lifestyle, the identification of which was not our primary goal when we selected subjects.

It is well acknowledged that selecting an adequate control group for any type of “expert” subpopulation poses a major challenge [33]. For example, instead of using a group of passive individuals, one could use a group that would also be considered expert, but in a different domain. By this, the particularities of the respective areas of expertise would have been compared. This evidently opens the door for many possible comparisons. The control group in our study was used to compare the effect of dancing. As dancing is coupled with physical exercise, our controls were selected to have neither experience with dance or sports. Further studies are required to test other groups comprised of individuals performing some type of sport activity in order to disentangle the effects of physical exercise hidden in dance. 

Subjects in the ED group performed better in most of the tasks investigated in this study. However, analysis of the individual IPs, which allowed comparison across all tests and subjects for the 5 domains (cognition, RT, posture and balance, motor performance, and tactile performance) showed a significantly better performance in the ED group with regard to RT and posture and balance only. Superior postural performance can be directly linked to the requirements imposed by dancing. A similar argument can be made for the finding of faster RTs in the ED group, which might be attributable to the requirements for both high attention and fast and well-coordinated motor responses. In contrast to the previously studied group of elderly amateur dancers [12], we found no differences between the ED and CG for performance measures related to the domains of cognition and hand-arm motor functions. On the other hand, this limited generalization is, to some extent, in agreement with recent studies that concluded that although high levels of expertise may have a positive impact on functions related specifically to that proficiency, other non-expert-related abilities of older experts were similar to those of nonexpert older adults [4]. 

In the previous study performed in a group of amateur dancers, we showed that the best performers of each task were present in both the dancing and the CG with similar frequency, but that the amateur dancing group lacked the number of poor performers that were frequently found within the control group [12]. Here, we showed that for non expertise-related domains such as tactile abilities, poor performers were equally present in both the ED and CG. These data led us to speculate that amateur and expert competitive dancers differ in that the latter focus on their area of expertise at the expense of other skills. Since dancing at a high maintained level of expertise requires extensive practice [15], competitive dancing requires substantial effort with regard to the traveling time and personal strain during competitions, which might counteract the positive impact of dancing that was observed in the amateur group.

Another line of argument for the limited positive impact of expertise at old age comes from functional imaging studies. It has been shown that learning piano playing in amateurs elicits stronger activation in a number of brain areas in comparison to the brain activation found in professional piano players, who practice playing to maintain high levels of expertise [34]. On the basis of these types of studies, it has been suggested that fewer cognitive resources are required for expert performance once an “automatic” and high-level stage is reached [35, 36]. It is therefore conceivable that the maintenance of high-level expertise has a lesser impact than the acquisition of new skills on the general fitness of older people. 

For many years, dance has been successfully established as a therapeutic tool in the elderly to improve cardiovascular parameters, muscle strength, and posture and balance [37, 38]. Our motivation for investigating the beneficial effects of dancing was triggered by the hypothesis that dancing can be regarded as an equivalent of enriched environmental conditions in human individuals [27], because of the unique combination of physical activity, rhythmic motor coordination, emotion, affection, balance, memory, social interaction, and acoustic stimulation. However, our data suggest that many of the features that play a crucial role in promoting positive effects in amateur dancing might be less relevant for expert dancers. This assumption is supported by the observation that the positive impact of expert competitive ballroom dancing was limited to expertise-related tasks such as posture and balance, or RTs [12]. Thus, years spent developing high-level expertise helps to maintain remarkable levels of posture-related performance even at an advanced age, which is in accordance with the notion that brain plasticity is operational in old age [27, 39]. 

Compared to activities such as exercising, walking, or playing an instrument, dance has the advantage of combining many diverse features including physical activity, social and emotional interaction, and sensory stimulation, each of which is well documented to have beneficial effects. Accordingly, there might also be many mechanisms mediating the positive outcomes of dancing. In healthy elderly individuals, physical fitness and cognitive performance are closely associated [5]. Consequently, many studies in the elderly have shown that improving aerobic capacity through physical exercise programs has beneficial effects on cognitive performance [6, 8, 9, 40–43]. While cardiovascular fitness might directly affect blood pressure and circulation, animal research on the effects of physical exercise suggests a crucial involvement of neurotrophins and other nerve growth factors [44, 45]. Use-dependent plasticity, synaptic efficacy, and the maintenance of synaptic connections are controlled and modulated by neurotrophins such as brain-derived neurotrophic factor (BDNF). BDNF levels increase by many factors such as physical activity and social interaction [44–47]. Housing animals under enriched environmental conditions, in particular, has been shown to increase neurotrophin gene expression, thus exerting neuroprotective functions [48–50]. Mild stress response in cells has been advocated as a major driving force for the upregulation of stress resistance genes and growth factors [51]. Interestingly, among the factors inducing mild stress are sensory stimulation, physical activity, and cognitive challenges, all of which are involved in dancing.

It must be recognized that the present study, as well as our previous study with amateur dancers [12], cannot resolve the query as to whether the superior performance of either the expert or amateur dancers is due to a group preselection of particularly fit subjects tending to engage in a regular schedule of year-long dancing or due to the dancing activity per se. It is therefore conceivable that intelligent people with better balance and faster RTs are those who are more likely to select dancing as a life-long avocation. However, both extreme standpoints seem unlikely, thus favoring a more intermediate stance. Recent data from an intervention study in a pre-/post-design showed that after a 6-month dance course, elderly participants improved in all tested aspects, including perception and cognition, similar to those described here [52]. These data show that irrespective of individual predispositions, dancing activities play a crucial role in mediating a wide range of beneficial effects, depending on the dose of exercise. Further studies are needed to investigate the contribution of individual predisposition and intervention.

Our data showed that a regular, multiyear schedule of expert and competitive ballroom dancing in a cohort of older individuals preserves posture and balance parameters to a remarkable extent and has a positive impact on RT. However, our results provided no evidence for more widespread beneficial effects on related domains such as tactile and cognitive performance. These findings suggest that not all doses of exercise are helpful for alleviating age-relating deterioration but hint at a more inverted U-shape, dose-response function with optimal ranges of intervention intensity required to have maximal beneficial effects. Accordingly, it might be important to adjust, depending on the individual level of activity and expertise, the challenges of intervention programs to maintain health and functional independence throughout life.

## 5. Conclusion

Given the dramatic demographic changes within industrialized countries characterized by an increasing probability of reaching old and very old age, there is an urgent need for measures permitting an independent lifestyle into old age. Since there is a close association between physical fitness and cognitive performance, a number of studies investigated the impact of interventional programs on the basis of dancing for the treatment of age-related functional degradation [37, 38, 53]. Our data showed that high levels of dancing expertise can be preserved up to a very old age, thereby maintaining remarkable levels of performance in expertise-related tasks. However, tasks outside these areas of expertise showed the age-related decline typically observed in aged-matched nonexperts.

## Figures and Tables

**Table 1:** Comparison of cognitive, posture, balance and sensorimotor status of ED and CG.

Variables	ED	(Range)	CG	(Range)	*Z*-score	*P* value	Effect size
Age (years)	71.18 ± 1.13	(66–77)	71.66 ± 1.11	(61*–*94)	−0.345	0.730	
Female (%)	54.55		78.94			0.121	
Education-level (schoolyears)	10.45 ± 0.39	(8–12)	10.16 ± 0.33	(6*–*13)	−0.696	0.486	
Everyday competence (ECQ)	10.62 ± 0.30	(9.13–12.28)	8.43 ± 0.34	(5.04*–*12.59)	−2.996	0.003	1.34
*Cognition*							
RSPM^1^	19.59 ± 0.75	(15–23.5)	15.39 ± 0.83	(7*–*28)	−2.776	0.006	1.04
Geriatric-concentration-test (AKT)	53.82 ± 0.40	(51.30–55)	53.49 ± 0.29	(47*–*55)	−4.997	≤0.001	0.21
*Reaction times*							
Multiple choice reaction times (ms), L	678.90 ± 21.22	(541.75–795.84)	780.30 ± 19.39	(581.01*–*1081.17)	−2.294	0.022	1.03
Multiple choice reaction times (ms), R	666.97 ± 20.07	(551.69–745.10)	760.32 ± 18.01	(580.51*–*1012.44)	−2.195	0.028	1.02
*Posture&balance*							
Romberg test (s), eyes open	60 ± 0.00	(60–60)	25.29 ± 4.40	(7.22*–*60)	−3.951	≤0.001	3.36
Romberg test (s), eyes closed	21.36 ± 6.45	(7.22–60)	14.44 ± 4.33	(2*–*42)	−1.250	0.211	0.38
Standing-turn (steps)	04.09 ± 0.71	(2–8)	5.64 ± 0.47	(3*–*8)	−1.765	0.078	0.78
Standing-turn (s)	01.91 ± 0.28	(1–4)	3.21 ± 0.33	(1.77*–*5.09)	−2.815	0.005	1.28
Up&go (s)	05.09 ± 0.21	(4–6)	7.42 ± 0.25	(5.54*–*8.54)	−3.819	≤0.001	3.02
*Motor Performance*							
*Hand-arm steadiness *							
Steadiness (error), L	0017 ± 4.91	(3–61)	15.82 ± 2.49	(0*–*54)	−0.394	0.694	0.07
Steadiness (error), R	15.64 ± 3.88	(3–45)	13.08 ± 2.72	(0*–*71)	−1.475	0.140	0.17
*Control Precision *							
Aiming (error), L	001.36 ± 0.34	(0–3)	1.00 ± 0.26	(0*–*5)	−1.584	0.113	0.26
Aiming (error), R	000.36 ± 0.20	(0–2)	1.87 ± 0.27	(0*–*6)	−2.808	0.005	1.18
Aiming (s), L	010.47 ± 0.67	(6.81–15.75)	11.18 ± 0.40	(7.19*–*18.37)	−0.760	0.447	0.30
Aiming (s), R	009.38 ± 0.57	(6.50–12.26)	10.66 ± 0.28	(7.96*–*17.11)	−1.889	0.059	0.71
Pin plugging (s), L	049.06 ± 1.93	(42.92–90.92)	48.84 ± 0.89	(37.64*–*62.45)	−0.086	0.932	0.49
Pin plugging (s), R	042.98 ± 1.58	(35.59–51.56)	48.31 ± 1.12	(37*–*64.64)	−2.343	0.019	0.87
*Rate of wrist movement *							
Tapping (hits), L	171.00 ± 6.88	(134–202)	154.63 ± 3.55	(102*–*207)	−1.902	0.057	0.73
Tapping (hits), R	184.18 ± 8.10	(134–215)	180.13 ± 3.97	(107*–*230)	−0.748	0.454	0.16
*Tactile performance *							
Touch-threshold (mN), LID	00.21 ± 0.04	(0.12–0.56)	0.22 ± 0.02	(0.08*–*0.54)	−0.775	0.438	0.08
Touch-threshold (mN), RID	00.32 ± 0.08	(0.12–0.95)	0.34 ± 0.04	(0.08*–*0.94)	−1.035	0.301	0.09
2-Point-discrimination-threshold (mm), LID	02.91 ± 0.18	(1.90–3.67)	3.45 ± 0.09	(2.20*–*4.64)	−2.515	0.012	0.96
2-Point-discrimination-threshold (mm), RID	02.98 ± 0.25	(1.86–4.55)	3.59 ± 0.07	(2.89*–*4.58)	−2.434	0.015	0.92

ED: expert dancer, CG: control group; L: left hand, R: right hand; LID: left index finger, RID: right index finger.

Values are means, SEM.

^1^Raven Standard Progressive Matrices, subset of 30 items.

**Table 2:** Indices of performance (IP) averaged across individual tasks describing cognition, reaction times, posture and balance, and motor, and tactile performance for both groups.

Domain	ED	(Range)	CG	(Range)	*Z*-score	*P* value	Effect size
Cognitive performance	0.73 ± 0.04	(0.38–1)	0.61 ± 0.04	(0–1)	−1.389	0.165	0.47
Reaction time	0.70 ± 0.02	(0.49–0.93)	0.52 ± 0.02	(0–0.87)	−3.462	0.001	1.02
Posture and balance	0.70 ± 0.04	(0–1)	0.37 ± 0.04	(0–1)	−5.599	≤0.001	1.26
Motor performance	0.74 ± 0.02	(0–1)	0.70 ± 0.01	(0–1)	−1.753	0.080	0.19
Tactile performance	0.56 ± 0.03	(0–0.99)	0.60 ± 0.02	(0–1)	−1.485	0.138	0.44

ED: expert dancer, CG: control group; Values are means, SEM.

## List of Abbreviations

ED: Expert dancer group
CG: Nondancer control group
RT: Reaction times
ECQ: Everyday competence questionnaire
RSPM: Raven Standard Progressive Matrices
AKT: Geriatric Concentration test
IP: Indices of performance
MMSE: Mini Mental Status Examination
IADL: Instrumental activities of daily living
2pd: 2-point discrimination threshold
LID: Left index finger
RID: Right index finger.
